# Mortality and biochemical recurrence after surgery, brachytherapy, or external radiotherapy for localized prostate cancer: a 10-year follow-up cohort study

**DOI:** 10.1038/s41598-022-16395-w

**Published:** 2022-07-22

**Authors:** José Francisco Suárez, Víctor Zamora, Olatz Garin, Cristina Gutiérrez, Àngels Pont, Yolanda Pardo, Alai Goñi, Alfonso Mariño, Asunción Hervás, Ismael Herruzo, Patricia Cabrera, Gemma Sancho, Javier Ponce de León, Víctor Macías, Ferran Guedea, Francesc Vigués, Manuel Castells, Montse Ferrer, Multicentric Spanish Group of Clinically Localized Prostate Cancer, Montse Ferrer, Olatz Garin, Àngels Pont, Yolanda Pardo, Jordi Alonso, Víctor Zamora, Ferran Guedea, Montse Ventura, Cristina Gutiérrez, Ferran Ferrer, Ana Boladeras, José Francisco Suárez, Manel Castells, Xavier Bonet, Mónica Ávila, Sergi Pastor, Carmen Bonet, Gemma Sancho, Javier Ponce de León, Joan Palou, Belén de Paula, Alai Goñi, Pablo Fernández, Ismael Herruzo, Asunción Hervás, Alfredo Ramos, Víctor Macías, Josep Solé, Marta Bonet, Alfonso Mariño, Patricia Cabrera, María José Ortiz

**Affiliations:** 1 Urology Department, Hospital Universitari de Bellvitge, L’Hospitalet de Llobregat, Barcelona, Spain; 2 Health Services Research Group, IMIM (Hospital del Mar Medical Research Institute), Doctor Aiguader, 88, Barcelona, Spain 08003; 3 CIBER en Epidemiología Y Salud Pública, CIBERESP, Madrid, Spain; 4 Universitat Autònoma de Barcelona, Cerdanyola del Vallès, Barcelona, Spain; 5 Universitat Pompeu Fabra (UPF), Barcelona, Spain; 6 Radiation Oncology Department, Institut Català d’Oncologia, L’Hospitalet de Llobregat, Barcelona, Spain; 7 Radiation Oncology Service, Fundación Onkologikoa - UGC Oncología Gipúzcoa, San Sebastián, Spain; 8 Radiation Oncology Department, Centro Oncológico de Galicia, A Coruña, Spain; 9 Radiation Oncology Department, Hospital Universitario Ramón y Cajal, Madrid, Spain; 10 Radiation Oncology Department, Hospital Regional Universitario de Málaga, Málaga, Spain; 11 Radiation Oncology Department, Hospital Universitario Virgen del Rocío, Sevilla, Spain; 12 Radiation Oncology Department, Hospital Santa Creu i Sant Pau, Barcelona, Spain; 13 Urology Department, Fundació Puigvert, Barcelona, Spain; 14 Radiation Oncology Department, Hospital Universitario y Politécnico La Fe, Valencia, Spain; 15 Institut Oncològic del Vallés (IOV), Sant Cugat del Vallès, Barcelona, Spain

**Keywords:** Outcomes research, Prostate, Prostate cancer

## Abstract

To compare the effectiveness at ten years of follow-up of radical prostatectomy, brachytherapy and external radiotherapy, in terms of overall survival, prostate cancer-specific mortality and biochemical recurrence. Cohort of men diagnosed with localized prostate cancer (T1/T2 and low/intermediate risk) from ten Spanish hospitals, followed for 10 years. The treatment selection was decided jointly by patients and physicians. Of 704 participants, 192 were treated with open radical retropubic prostatectomy, 317 with ^125^I brachytherapy alone, and 195 with 3D external beam radiation. We evaluated overall survival, prostate cancer-specific mortality, and biochemical recurrence. Kaplan–Meier estimators were plotted, and Cox proportional-hazards regression models were constructed to estimate hazard ratios (HR), adjusted by propensity scores. Of the 704 participants, 542 patients were alive ten years after treatment, and a total of 13 patients have been lost during follow-up. After adjusting by propensity score and Gleason score, brachytherapy and external radiotherapy were not associated with decreased 10-year overall survival (aHR = 1.36, p = 0.292 and aHR = 1.44, p = 0.222), but presented higher biochemical recurrence (aHR = 1.93, p = 0.004 and aHR = 2.56, p < 0.001) than radical prostatectomy at ten years of follow-up. Higher prostate cancer-specific mortality was also observed in external radiotherapy (aHR = 9.37, p = 0.015). Novel long-term results are provided on the effectiveness of brachytherapy to control localized prostate cancer ten years after treatment, compared to radical prostatectomy and external radiotherapy, presenting high overall survival, similarly to radical prostatectomy, but higher risk of biochemical progression. These findings provide valuable information to facilitate shared clinical decision-making.

**Study identifier at ClinicalTrials.gov**: NCT01492751.

## Introduction

In the European Union, prostate cancer was the most frequently diagnosed tumor, and the third leading cause of death of all cancers in men^1^. The EUROCARE-V study^2^ showed that the age-standardized 5-year relative survival improved from 73% for the period 1999–2001 to 82% in 2005–2007. In general, prostate cancer incidence has increased, while its mortality has decreased in most countries^3^ since prostate-specific antigen (PSA) detection. Currently, most prostate cancer patients are diagnosed in localized stages^4^ and managed mainly through active monitoring, radical prostatectomy or radiotherapy, which involves either external radiation therapy or brachytherapy^5^.

The main randomized controlled trial of curative intention treatments for localized prostate cancer is ProtecT (Prostate Testing for Cancer and Treatment)^6,7^. It showed no differences on overall survival and prostate cancer-specific mortality at ten years of follow-up^6^ for radical prostatectomy, external radiotherapy and active monitoring, but higher rates of disease progression and metastases for men in active monitoring. However, brachytherapy was not assessed in the ProtecT trial^6,7^, and the observational studies comparing it with the other radical treatments at long term are scarce.

Three studies reporting comparative effectiveness research on localized prostate cancer patients^8–10^ showed that brachytherapy and external radiotherapy were associated with lower long-term overall survival than radical prostatectomy, but not higher prostate cancer-specific mortality. The first study, published in 2012, reported results for the whole sample (n = 10,429)^8^ and for patients without comorbidities^11^, with a median follow-up among survivors of 5.6 years. The other two have been published recently, one focused on patients older than 70 years with median follow-ups from 5.3 to 7.5 years^9^, and one on patients with intermediate risk tumors with a median follow-up of 10 years^10^. Only the latter reported biochemical recurrence, showing better results for brachytherapy than for radical prostatectomy. In contrast, a meta-analysis^12^ synthesizing results of biochemical recurrence from studies with a median follow-up of 5 years or shorter obtained no significant difference between brachytherapy and radical prostatectomy.

Therefore, comparative effectiveness research on survival and biochemical recurrence among radical prostatectomy, brachytherapy and external radiotherapy at long term is needed. The aim of this study was to compare the effectiveness among treatments with curative intention in terms of overall survival, prostate cancer-specific mortality and biochemical recurrence on patients with localized prostate cancer ten years after radical prostatectomy, brachytherapy or external radiotherapy.

This is a prospective cohort analysis of data from a project named “Multicentric Spanish Study of Clinically Localized Prostate Cancer”, composed of men diagnosed with clinically localized prostate cancer who had been followed prospectively (ClinicalTrials.gov Identifier: NCT01492751). Results of this cohort have been previously described in terms of impact of treatments’ side effects measured with patient-reported outcomes, which were the primary endpoints (at two^13^, three^14^, five^15^ and ten years^16^ of follow-up), but no report on mortality and biochemical recurrence has been published. The latter were a priori decided as secondary outcomes.

## Methods

### Patients

This was a prospective observational study of a cohort of men diagnosed with clinically localized prostate cancer and treated with radical prostatectomy (n = 192), brachytherapy (n = 317), or external radiotherapy (n = 195). The study was approved by the ethics review boards of the participating hospitals, and written informed consent was obtained from patients, following the 2000 revision of the Helsinki Declaration.

Study details have been described elsewhere^13–16^. Briefly, participants in the “Multicentric Spanish Study of Clinically Localized Prostate Cancer” were consecutively recruited from ten Spanish hospitals in 2003–2005. Eligible patients had a clinical stage T1 or T2 and were treated with radical retropubic prostatectomy, external radiotherapy or brachytherapy. Initial exclusion criteria of the cohort^13,14^ were: treated in other hospitals than the participating centers, and previous prostate transurethral resection. According to advances in scientific evidence^17^, high risk was added to the exclusion criteria in 2011^15,16^. The definition by D’Amico et al.^18^ was used to classify patients into risk groups: T1c or T2a, PSA ≤ 10 ng/mL and Gleason ≤ 6 for low risk; and T2b, PSA 11–20 ng/mL or Gleason 7 for intermediate risk. Out of the 841 patients recruited, 61 were high-risk patients, 44 did not meet other inclusion criteria, 18 were transferred to other hospitals before treatment, and 14 refused to participate, giving a total of 704 participants^16^, which was the final sample included in all analyses.

### Treatment

The decision regarding treatment selection was made jointly by patients and physicians. Patients who chose surgery underwent open radical retropubic prostatectomy. Nerve-sparing techniques were applied at the discretion of the operating surgeon on 27% of the patients. The brachytherapy group received interstitial low dose radiotherapy alone with ^125^I. The prescription dose was 144 Gy to the reference isodose (100%) according to the Task Group 43^19^. The median dose of D90 and V100% was 154 Gy (range 97–205.3) and 94% (range 60–100), respectively. External beam radiation was performed using the 3-D conformal technique (prostate planning target volume in 1.8 to 2.0 Gy daily fractions). Seminal vesicles and regional lymphatics were also contoured if risk of involvement was suspected. Mean dose was 74 Gy (range 65–78), with only seven patients receiving < 70 Gy.

### Clinical evaluations

Demographic and clinical characteristics at baseline were recorded at clinical sites, including age, PSA, Gleason score, and risk group as defined by D’Amico et al.^18^. Serum PSA levels were measured at follow-up visits, every 6 months for the first 2 years and annually thereafter. Biochemical recurrence was defined as: (a) an initial PSA value after radical prostatectomy > 0.2 ng/mL (used to define the time of recurrence) followed by a second confirmatory PSA value > 0.2 ng/mL^20^, as per the American Urological Association criteria; or (b) a PSA increase of > 2 ng/ml from the nadir after radiotherapy or a need for salvage therapy^21^, following the American Society for Radiation Oncology criteria.

### Sample size calculation

The sample size of this study was calculated to detect differences on patient-reported outcomes between groups^13^. The statistical power for comparing overall survival and biochemical recurrence between brachytherapy and radical prostatectomy (with 317 and 192 patients, respectively) was 0.8 or higher, using a 2-sided long rank test with type 1 error of 5%. The statistical power drops to 0.7 for comparing prostate cancer-specific mortality due to the small number of events.

### Mortality

Vital status, date and cause of death were confirmed by linkage with the National Institute of Statistics^22^ up to April 2017. Codes in the 11th International Classification of Diseases for malignant prostate tumor (2C82) and prostate tumor of uncertain or unknown behavior (2F97&XA63E5) were considered as prostate cancer-specific mortality.

### Statistical analysis

The characteristics of the patients were described and compared among treatment groups after adjusting by the previously developed propensity scores^15^. Briefly, to account for treatment selection bias, a multinomial logistic regression model was constructed to estimate the conditional probability of receiving a treatment, given measured covariates, with c-statistics ranging from 0.81 to 0.92 among treatment groups. This model included socio-demographic variables, prostate cancer characteristics, number of chronic conditions, medication, tobacco consumption, and family cancer history. Unlike propensity score matching, this statistical approach includes all the study participants and makes a comparison amongst several groups more feasible^23^.

To analyze overall survival, prostate cancer-specific mortality, and biochemical recurrence, the time periods used were from date of treatment to date of the event or date of the latest available information. Patients with no biochemical recurrence were censored at the last follow-up in which PSA information was available. Summary statistics and 95% confidence intervals (95% CI) are reported according to treatment group. To compare overall survival among the three treatment groups, Kaplan–Meier curves and Cox proportional-hazards regression models were constructed using follow-up data until year 11 after treatment. To compare prostate cancer-specific mortality and biochemical recurrence, cumulative incidence functions were plotted and Fine and Gray models^24^ with proportional hazards for the subdistribution of death as a competing risk were constructed: death from all other causes in the prostate cancer-specific mortality model and death from all causes in the biochemical recurrence model. Nested models were constructed by adding variables sequentially: model 1 only with treatment effect; model 2 adjusting by propensity scores; and model 3 adding the clinical variables which remained unbalanced among treatment groups after propensity scores adjustment. Proportional hazards assumptions were tested using weighted residuals^25^. All available data were included in the analysis, and any missing follow-up outcome data were assumed to be missing at random. R version 4.1.1 was used for survival analyses.

### Ethics statement

The present study has been reviewed and approved by the Institutional Review Board at the Hospital del Mar Medical Research Institute, in accordance with the 2000 Declaration of Helsinki. The study was also approved by the ethics committees from the participating sites: Institut Català d’Oncologia, Hospital Universitari de Bellvitge, Hospital de la Santa Creu i Sant Pau, Fundació Puigvert, Institutu Onkologikoa de Gipúzkoa, Hospital Regional Universitario de Málaga, Hospital Universitario Ramón y Cajal, Hospital Universitari General de Catalunya and Centro Oncológico de Galicia and Hospital Universitario Virgen del Rocío. Written informed consent was provided by all subjects when they were enrolled.

## Results

### Pretreatment baseline characteristics

Table 1 shows baseline characteristics of the patients. Most of participants were aged 66 years or older, presented PSA below 10 ng/mL, Gleason score equal or lower than six, clinical stage ≤ T2a, and they were classified as D’Amico low risk group. After propensity scores adjustment, no statistically significant differences were observed among treatment groups, except for Gleason score, which ranged from 96.3% of patients with values equal or lower than six in brachytherapy to 90% in external radiotherapy (p-value = 0.038). Median (interquartile range—IQR) follow-up for vital status adjusted by propensity scores was 10 years (10.0–10.0) in the three treatment groups, while median and IQR follow-up for biochemical recurrence was 10 years (9.1–10.0) in radical prostatectomy group, 9.9 years (9.1–10.0) in brachytherapy, and 10.0 (8.6–10.0) in external radiotherapy.

**Table 1:** Baseline characteristics by treatment: unadjusted n and adjusted percentages with propensity scores, and median [IQR] follow-up.

	All	Radical prostatectomy	Brachytherapy	External radiotherapy	Adjusted p-value^a^
Participants	704	192	317	195	
**Unadjusted n (adjusted %)**					
**Age**					0.484
≤ 65 years	265 (33.0%)	114 (32.8%)	113 (33.8%)	38 (31.7%)	
66 – 70 years	222 (40.2%)	61 (47.6%)	100 (37.4%)	61 (37.6%)	
≥ 71 years	216 (26.8%)	16 (19.6%)	104 (28.8%)	96 (30.8%)	
Missing	*1 (0.1%)*	*1 (0.5%)*	*0*	*0*	
**PSA**					0.590
< 10	603 (98.0%)	155 (92.4%)	294 (89.6%)	154 (92.4%)	
[10, 20]	100 (1.9%)	36 (7.6%)	23 (10.4%)	41 (7.6%)	
Missing	*1 (0.1%)*	*1 (0.5%)*	*0*	*0*	
**Gleason score**					0.038
≤ 6	563 (93.7%)	114 (92.6%)	308 (96.3%)	141 (90.0%)	
7	139 (6.3%)	76 (7.4%)	9 (3.7%)	54 (10.0%)	
Missing	*2 (0.3%)*	*2 (1.0%)*	*0*	*0*	
**Clinical T Stage**					0.439
≤ T2a	651 (97.6%)	175 (98.6%)	307 (96.8%)	169 (97.9%)	
T2b	47 (2.4%)	13 (1.4%)	9 (3.2%)	25 (2.1%)	
Missing	*6 (0.9%)*	*4 (2.1%)*	*1 (0.3%)*	*1 (0.5%)*	
**Risk group**					0.756
Low	482 (86.5%)	91 (88.3%)	283 (85.0%)	108 (87.1%)	
Intermediate	222 (13.5%)	101 (11.7%)	34 (15.0%)	87 (12.9%)	
**Neoadjuvant hormonal treatment**					0.764
No	521 (81.4%)	175 (78.6%)	212 (82.7%)	134 (82.0%)	
Yes	183 (18.6%)	17 (21.4%)	105 (17.5%)	61 (18.4%)	
**Adjusted median [IQR] follow-up, years**					
Vital status		10.0 [10.0, 10.0]	10.0 [10.0, 10.0]	10.0 [10.0, 10.0]	
Biochemical recurrence		10.0 [9.0, 10.0]	9.9 [9.1, 10.0]	10.0 [8.6, 10.0]	

^a^p-value was obtained with likelihood ratio test. Follow-up was calculated in years.

*IQR* interquartile range, *PSA* prostate-specific antigen.

### Flow-chart

The flow chart (Supplementary Fig. S1) shows that of the 704 participants, 542 patients were alive after ten years of follow-up, and a total of 13 patients have been lost during follow-up: three in the radical prostatectomy group, eight in the brachytherapy group and two in external radiotherapy. The number of patients with missing clinical data for biochemical recurrence at 10 years was 31 (19.3%) in radical prostatectomy, 62 (25.8%) in brachytherapy and 39 (27.7%) in external radiotherapy.

### Overall survival

**Figure 1:**
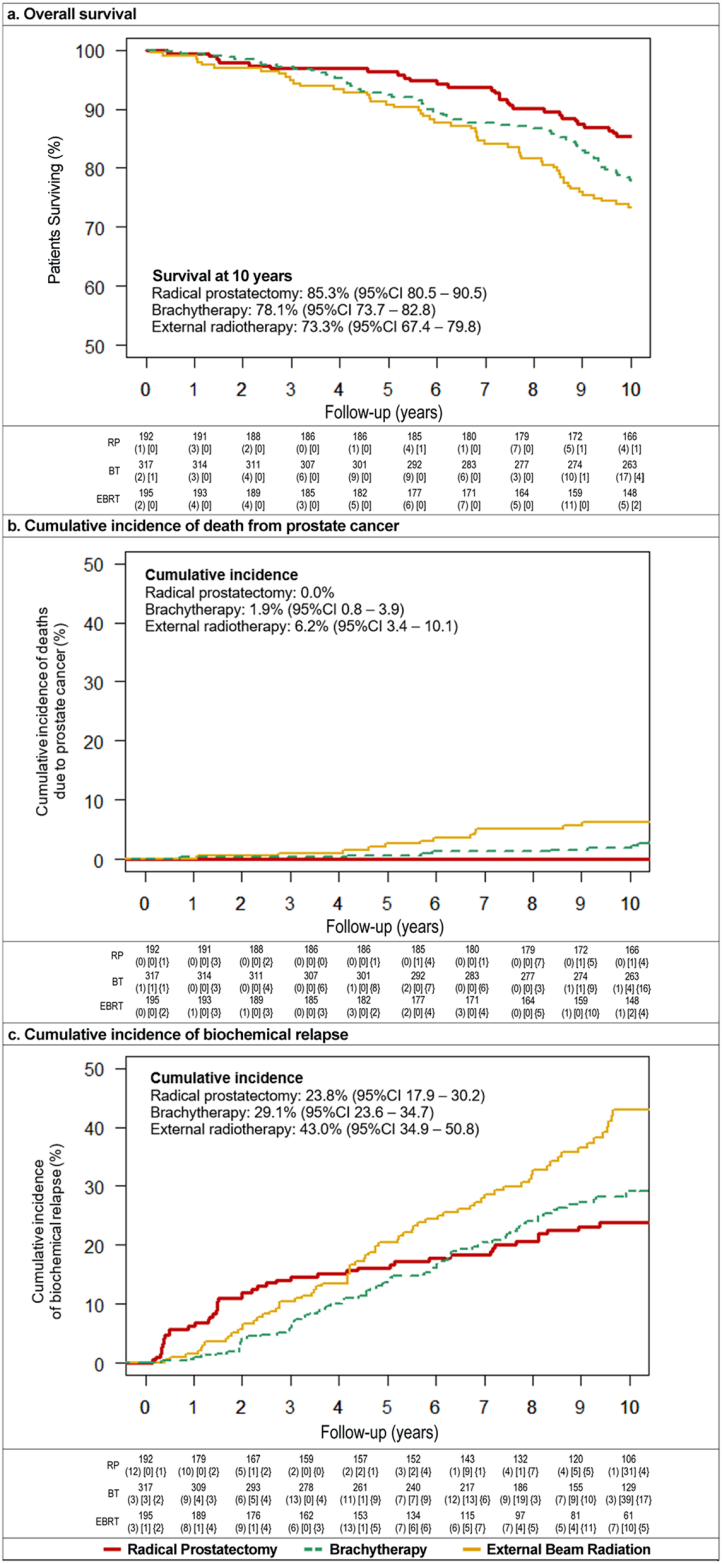
Kaplan–Meier plots among radical prostatectomy (red line), brachytherapy (green line) and external radiotherapy (yellow line). *BT* Brachytherapy, *EBRT* External Beam Radiotherapy, *RP* Radical Prostatectomy. Numbers represent: patients at the beginning of the year; (patients with the event of interest); [patients lost to follow-up]; and {patients at competing risk}.

Figure 1a shows Kaplan–Meier curves of overall survival at ten years after treatment, which was 85.3% (95% CI 80.5–90.5) for patients who underwent radical prostatectomy, 78.1% (95% CI 73.7–82.8) for those in the brachytherapy group, and 73.3% (95% CI 67.4–79.8) for those treated with external radiotherapy. Table 2 shows statistically significant differences in overall survival among treatment groups in Model 1. After adjusting by propensity scores (Model 2), the significant differences disappeared: hazard ratios were 1.32 (95% CI 0.75–2.33) for brachytherapy and 1.40 (95% CI 0.78–2.51) for external radiotherapy, compared to radical prostatectomy (reference group). After adding the Gleason score in model 3, differences on hazard ratios among treatment groups remained not significant.

**Table 2:** Cox proportional-hazards regression models for overall survival, prostate cancer-specific mortality and biochemical recurrence (model 1), adjusted by propensity scores (model 2), and Gleason score (model 3).

	Model 1		Model 2		Model 3	
	HR (95% CI)	p-value	HR (95% CI)	p-value	HR (95% CI)	p-value
**Overall Survival**						
Radical prostatectomy	Ref		Ref		Ref	
Brachytherapy	1.67 (1.10—2.52)	0.015	1.32 (0.75 – 2.33)	0.342	1.36 (0.77—2.42)	0.292
External radiotherapy	1.98 (1.28—3.06)	0.002	1.40 (0.78—2.51)	0.259	1.44 (0.80—2.59)	0.222
Propensity score_a_						
Propensity score_b_			1.50 (0.64 – 3.52)	0.347	1.44 (0.57—3.61)	0.442
Gleason score			2.36 (0.96 – 5.78)	0.061	2.33 (0.95—5.74)	0.066
≤ 6					Ref	
7					0.96 (0.60—1.55)	0.882
**Prostate Cancer-Specific Mortality**^**a**^						
Radical prostatectomy	Ref		Ref		Ref	
Brachytherapy	5.55 (0.71—43.24)	0.100	4.45 (0.69 – 28.71)	0.120	4.41 (0.69—28.29)	0.120
External radiotherapy	12.23 (1.61—92.95)	0.016	9.46 (1.55 – 57.79)	0.015	9.37 (1.53—57.21)	0.015
Propensity score_a_			1.54 (0.15 – 16.05)	0.720	1.58 (0.17—14.45)	0.690
Propensity score_b_			1.85 (0.26 – 12.98)	0.540	1.85 (0.27—12.52)	0.530
Gleason score						
≤ 6					Ref	
7					1.04 (0.31—3.47)	0.960
**Biochemical recurrence**^**a**^						
Radical prostatectomy	Ref		Ref		Ref	
Brachytherapy	1.14 (0.78—1.67)	0.490	1.89 (1.20 – 2.95)	0.006	1.93 (1.23—3.03)	0.004
External radiotherapy	1.76 (1.20—2.59)	0.004	2.50 (1.60 – 3.89)	< 0.001	2.56 (1.64—3.98)	< 0.001
Propensity score_a_			0.34 (0.17 – 0.67)	0.002	0.36 (0.17—0.75)	0.006
Propensity score_b_			0.52 (0.26 – 1.05)	0.067	0.52 (0.26—1.06)	0.074
Gleason score						
≤ 6					Ref	
7					1.05 (0.86—1.29)	0.610

*95% CI* 95% Confidence Interval, *HR* Hazard Ratio, *Propensity Score*_*a*_ adjustment in brachytherapy with the entire cohort, *Propensity Score*_*b*_ adjustment in external radiotherapy with the entire cohort.

^a^Adjusted by competing risk.

### Prostate cancer-specific mortality

Figure 1b shows Kaplan–Meier curves of death from prostate cancer up to ten years after treatment. The cumulative incidence was 0.0% in the radical prostatectomy group, 1.9% (95% CI 0.8–3.9) for brachytherapy, and 6.2% (95% CI 3.4–10.1) for external radiotherapy. Table 2 shows statistically significant differences among treatment groups for Model 1, but the hazard ratio remained statistically significant only for external radiotherapy after adjusting by propensity scores (Model 2 and 3). Compared to the radical prostatectomy group, in Model 3 the hazard ratio was 9.37 (95% CI 1.53–57.21, p = 0.015) for external radiotherapy, and 4.41 (95% CI 0.69–28.29, p = 0.120) for brachytherapy, but it was not statistically significant.

### Biochemical recurrence

Figure 1c shows Kaplan–Meier curves of biochemical recurrence. Ten years after treatment, the cumulative incidence was 23.8 (95% CI 17.9–30.2) in the radical prostatectomy group, 29.1% (95% CI 23.6–34.7) for the brachytherapy group and 43.0% (95% CI 34.9–50.8) in the external radiotherapy group. Results of multivariable Cox regression models (Table 2) showed that, after adjustment for propensity score, the risk of biochemical recurrence (compared to radical prostatectomy) was significantly higher for patients receiving either brachytherapy or external radiotherapy, with hazard ratios of 1.93 (95% CI 1.23–3.03) and 2.56 (95% CI 1.64–3.98) in Model 3. Biochemical recurrence appeared at a median of 4.4 years after treatment: 2.0 years (IQR 0.6–5.7) for radical prostatectomy, 4.8 years (IQR 3.0–6.9) for brachytherapy and 4.6 years (IQR 2.7–7.2) for external radiotherapy.

## Discussion

This study comparing oncological outcomes after ten years of the three most established treatment options for localized prostate cancer patients shows that brachytherapy and external radiotherapy were not associated with decreased 10-year overall survival, but presented higher biochemical recurrence compared with radical prostatectomy in men with clinically localized prostate cancer. There have been other studies showing long-term results^8–10^ but, to our knowledge, this is the first one evaluating mortality, together with biochemical recurrence, in patients at low and intermediate prostate cancer risk.

Our results showing no differences in overall survival between radical prostatectomy and radiotherapy groups at ten years were consistent with the ProtecT randomized clinical trial all-cause mortality results between prostatectomy and external radiotherapy^6^. However, our findings differ from non-randomized studies, all of which showed significantly higher risk of death for radiotherapy groups than for radical prostatectomy: aHR of 1.7–1.5^8^, 1.8^9^, and 1.6^10^ for brachytherapy; and aHR of 1.7–1.5^8^, 1.95^9^, and 1.9^10^ for external radiotherapy. This higher mortality risk in radiation groups could be explained by differences in the sample characteristics, such as age^9^ or comorbidities^11^, between these studies and ours. In fact, the consistent demonstration in those studies of no higher prostate cancer‐specific mortality^8–10^ suggests that differences in all-cause mortality are associated with patients’ factors unrelated to prostate cancer. On the other hand, differences between surgical and radiation primary therapies in combined treatment with adjuvant androgen deprivation, associated with certain complications^26,27^, could partially explain this higher mortality risk.

We found relevant differences in prostate cancer-specific mortality among treatments at ten years of follow-up. Our results show higher statistically significant risk for external radiotherapy (aHR = 9.37, p = 0.015), but it was not significant for brachytherapy (aHR = 4.41, p = 0.120), compared to radical prostatectomy. In contrast, all the previous observational studies showed no significant higher risk of death due to prostate cancer, with smaller hazard ratios for brachytherapy (aHR of 1.3^9^, 1.4^10^, and 2.3 for low and 0.6 for intermediate tumoral risk^8^) and external radiotherapy (aHR of 1.7^9^, 1.1^10^, and 1.8 for both tumoral risk groups^8^). This difference in the magnitude of risk is explained by the remarkably low prostate cancer-specific mortality within the radical prostatectomy group in our study, close to zero, compared to the others: 1.8%^8^, 1.2%^9^, and 3.4%^10^. Underestimation by chance cannot be discarded, since there was only one death caused by prostate cancer among the 192 surgery patients, which occurred ten years and nine months after treatment. Differential misclassification between death by prostate cancer and death by other causes among treatment groups was minimized by obtaining cause of death through linkage with the National Institute of Statistics^22^.

In our study, brachytherapy showed lower risk of biochemical recurrence than external radiotherapy (29.1% vs 43.0%), consistently with previous findings (29% vs 67%)^28^, but higher risk than radical prostatectomy (29.1% vs 23.8%; and aHR of 1.93, in favor of radical prostatectomy). This latter finding clearly contrasts with previous publications^10,12^. On one hand, the meta-analysis of 8,385 patients^12^ obtained an odds ratio of 1.24 (95% CI 0.91–1.68) in favor of brachytherapy, but with considerable heterogeneity (I^2^ = 77%). On the other hand, the most recent observational study focused on intermediate risk^10^ also showed at ten years of follow-up a lower biochemical recurrence rate in brachytherapy (19.8%) than in radical prostatectomy (42.9%) after propensity scores adjustment. This rate in their radical prostatectomy group is twice that of our cohort (23.8%), mainly composed by low-risk prostate cancer patients. This suggests a relationship between tumoral risk and biochemical recurrence among surgery patients. In fact, in our study the rate for patients treated with radical prostatectomy was 12.1% in low risk and 32.7% in intermediate risk, the latter closer to the rate found in the Goy et al. study^10^.

The main limitation of this study is its observational design. The main concern is a possible treatment selection bias where, for example, brachytherapy is preferentially prescribed to patients with lower tumor risk, and surgery to younger patients^13–15^. In our cohort, the propensity scores balanced treatment selection bias^15^, except for Gleason score. Therefore, we constructed a final model adjusted with propensity scores and Gleason score. Furthermore, these results are consistent with those obtained through traditional adjustment in the Cox models (Supplementary Table S1). Secondly, results of prostate cancer-specific mortality should be interpreted with caution due to the low number of deaths attributed to prostate cancer in our cohort, which affects the statistical power. Thirdly, the results on biochemical recurrence could have been affected by missing data, since there were 132 patients (24.4%) with no PSA at 10 years of follow-up. Missing data ranged from 19.3% in radical prostatectomy to 27.7% in external radiotherapy, therefore the differences between radical prostatectomy and radiotherapy groups might be underestimated. However, no statistically significant differences were found between patients with and without PSA data at 10 years of follow-up (data not shown). Finally, the treatments were applied over one decade ago; since then, diagnostic techniques and treatments for prostate cancer have evolved. For instance, most new high-tech radiotherapy modalities seem to enhance patient survival and therapeutic gain^29^, but hypofractionation is still controversial^30^; therefore, further research in patients undergoing new modalities of treatment is needed.

The strengths of the study are certainly the large number of patients who completed the 10-year follow-up, which provides robust evidence on overall mortality and biochemical recurrence, especially for patients who underwent brachytherapy. Moreover, confirmation of all-cause mortality and prostate cancer-specific mortality with the National Institute of Statistics (INE) provided reliable and unbiased data for date and cause of death. Finally, our study’s outcome collection was in accordance with the current recommendations of the International Consortium for Health Outcomes Measurement (ICHOM) for localized prostate cancer, which were developed in 2015^31^.

Further than survival and biochemical recurrence, it is important to consider other outcomes for treatment selection in localized prostate cancer. Acute complications and patient-reported outcomes are also included in ICHOM’s recommendations^31^. The results on survival and biochemical recurrence of the present study need to be balanced with results on patient-reported outcomes previously published^16^, showing that brachytherapy is the treatment option that causes the least negative impact. However, no single treatment can be considered the preferred strategy for managing all patients, and each treatment decision should be made jointly by patients and physicians^5^.

In conclusion, novel long-term results are provided on the effectiveness of brachytherapy to control localized prostate cancer during ten years after treatment, compared to radical prostatectomy and external radiotherapy. It presents high overall survival similarly to radical prostatectomy, but higher risk of biochemical progression. These results provide patients, clinicians, and health planners with valuable information, considered jointly with the other relevant outcomes, to make evidence-based decisions and facilitate shared clinical decision-making.

## Supplementary Information

Supplementary Information.

## Data Availability

The datasets generated and analysed during the current study are not publicly available due to institutional policies for sensitive data, but a de-identified version is available from the corresponding author upon reasonable request.
