# Supplementary material for: Mortality and biochemical recurrence after surgery, brachytherapy, or external radiotherapy for localized prostate cancer: a 10-year follow-up cohort study
Source: Sci Rep. 2022 Jul 22;12:12589. doi: 10.1038/s41598-022-16395-w (PMC9307750; doi:10.1038/s41598-022-16395-w)
Supplement: Supplementary file 1 — Supplementary Information. [file 41598_2022_16395_MOESM1_ESM.pdf]

Supplementary Fig S1. Flow chart of patients from recruitment to ten years after treatment: overall survival, prostate cancer-specific mortality and biochemical recurrence.

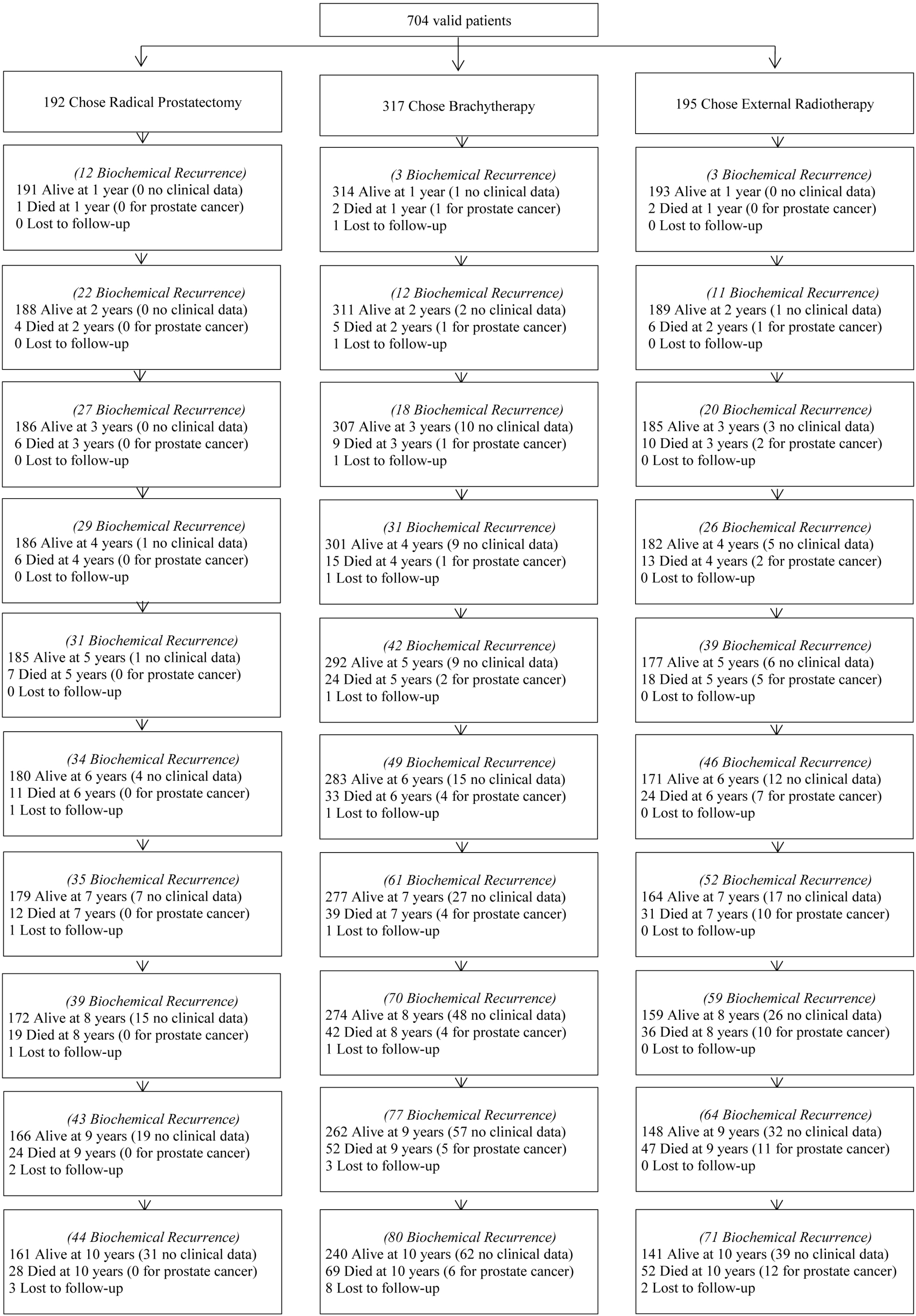

Supplementary Table S1. Cox proportional-hazards regression models for all-cause mortality, prostate cancer-specific mortality and biochemical recurrence, adjusted by age, number of chronic conditions, PSA, Gleason score and tumoral stage.

	**All-cause mortality**		**Prostate cancer-specific mortality**		**Biochemical recurrence**	
	**HR (95% CI)**	**p-value**	**HR (95% CI)**	**p-value**	**HR (95% CI)**	**p-value**
**Radical Prostatectomy**	Ref.		Ref.		Ref.	
**Brachytherapy**	1.23 (0.78 - 1.94)	0.373	4.55 (0.69 - 30.21)	0.120	1.46 (0.98 - 2.16)	0.062
**External Radiotherapy**	1.24 (0.77 - 2.01)	0.377	8.48 (1.18 - 61.03)	0.034	1.96 (1.28 - 3.01)	0.002
**Age**	1.08 (1.05 - 1.11)	0.000	1.04 (0.95 - 1.13)	0.400	0.97 (0.95 - 0.99)	0.012
**Number of chronic conditions**	1.13 (1.02 - 1.24)	0.016	1.23 (0.93 - 1.64)	0.150	0.96 (0.86 - 1.06)	0.410
**PSA**	1.00 (0.95 - 1.06)	0.995	1.03 (0.85 - 1.23)	0.780	1.09 (1.04 - 1.14)	0.001
**Gleason total**	0.93 (0.79 - 1.10)	0.419	0.91 (0.58 - 1.44)	0.690	1.10 (0.90 - 1.33)	0.350
**T**						
**T1**	Ref.		Ref.		Ref.	
**T2**	0.88 (0.62 - 1.26)	0.486	1.30 (0.51 - 3.35)	0.580	1.27 (0.92 - 1.76)	0.140
**Missing T**	2.92 (0.40 - 21.45)	0.292				
**Propensity Score$_a$**	1.50 (0.64 – 3.52)	0.347	1.54 (0.15 – 16.05)	0.720	0.34 (0.17 – 0.67)	0.002
**Propensity Score$_b$**	2.36 (0.96 – 5.78)	0.061	1.85 (0.26 – 12.98)	0.540	0.52 (0.26 – 1.05)	0.067

95% CI=95% Confidence Interval; HR=Hazard Ratio; Propensity Score$_a$=adjustment in brachytherapy with the entire cohort; Propensity Score$_b$=adjustment in external radiotherapy with the entire cohort.
